# Electrochemical Sensors Based on Self-Assembling Peptide/Carbon Nanotube Nanocomposites for Sensitive Detection of Bisphenol A

**DOI:** 10.3390/s24051465

**Published:** 2024-02-23

**Authors:** Yuhang Zhang, Tingting Shao, Hangyu Zhang

**Affiliations:** 1 School of Biomedical Engineering, Faculty of Medicine, Dalian University of Technology, Dalian 116024, China; 2 Liaoning Key Lab of Integrated Circuit and Biomedical Electronic System, Dalian University of Technology, Dalian 116024, China

**Keywords:** sensor, carbon nanotube, self-assembling peptide, electrochemical, bisphenol A

## Abstract

In this study, a cationic amphiphilic self-assembling peptide (SAP) Z23 was designed, and a simple bisphenol a (BPA) sensor, based on SAP Z23/multiwalled carbon nanotubes (Z23/MWCNTs) composite, was successfully fabricated on the surface of a glassy carbon electrode (GCE). The composite material was formed by π-π stacking interaction between the aromatic group on the hydrophobic side of Z23 and the side-wall of MWCNTs, with the charged hydrophilic group of Z23 exposed. During the electrocatalytic process of BPA, a synergistic effect was observed between Z23 and MWCNTs. The current response of the sensor based on composite material was 3.24 times that of the MWCNTs-modified electrode, which was much higher than that of the peptide-based electrode. Differential pulse voltammetry (DPV) was used to optimize the experimental conditions affecting the analytical performance of the modified electrode. Under optimal conditions, the linear range of the sensor was from 10 nM to 100 μM by amperometric measurement with sensitivity and limit of detection (LOD) at 6.569 μAμM^−1^cm^−2^ and 1.28 nM (S/N = 3), respectively. Consequently, the sensor has excellent electrochemical performance and is easy to fabricate, making it a good prospect in the field of electrochemical detection in the future.

## 1. Introduction

BPA is well-known to be a typical endocrine disruptor. It causes adverse health effects by mimicking the human endogenous hormones and is correlated to a multitude of diseases, such as reproductive system diseases, cancer, and cardiovascular disease [1]. BPA is primarily used in the production of polymer materials such as epoxy resins, polycarbonate and polysulfone resins [2]. At present, there are several methods to determine its concentration, including liquid chromatography–mass spectrometry (LC–MS) [3], antibody-based surface plasmon resonance (SPR) [4], Förster resonance energy transfer [5], capacitive aptamer detection [6], and electrochemical sensing. Due to the high cost of instruments, tedious experimental procedures (including repeated incubation and cleaning steps), and long sample processing time, most of them are unsuitable for on-site environmental monitoring and food safety testing. Electrochemical methods can effectively detect BPA, but they face challenges with low sensitivity and poor reproducibility. A practical method to overcome these obstacles is electrode modification, which can significantly increase the electron transfer rate on the electrode surface and reduce overpotential. Various nanostructures such as graphene [7], mesoporous silica [8], nanoparticles [9], and carbon materials [10,11] have been successfully used to modify bare electrodes.

Carbon nanotubes attract extensive attention due to their unique nanostructures and electrical properties. Functionalized-multiwalled carbon nanotubes (f-MWCNTs) are the hydrophilic oxidative derivative of MWCNTs and owe their functionalities to the presence of oxygen [12]. Carbon nanotubes have been crucial in the development of chemical and biosensors due to their nanometer scale, high mechanical strength, and excellent electron conductivity. Since their discovery, they have played an important role in this field [13]. Unmodified carbon nanotubes aggregate in most solvents because of strong Van der Waals forces, requiring external functionalization to create a physical or electrostatic barrier to improve their solubility [14]. Common methods include physical blending [15], in situ polymerization, covalent side-wall coupling [16,17], and non-covalent outer corner interactions [18,19], but their dispersion efficiency is low, and the steps are cumbersome and not eco-friendly. Hydroxyl or carboxyl functionalized carbon nanotubes with better solubility can be applied to modify electrodes. However, they may lead to insufficient detection limits and a narrow linear response range.

Self-assembly is a widespread phenomenon in life activities. By rearranging the sequence of the peptide molecule and altering the external environment, SAP molecules are spontaneously driven by the synergistic action of hydrogen bonding, electrostatic interaction, hydrophobic interactions, and π-π stacking between benzene rings to form stable assemblies with specific structures and functions [20,21]. Compared with other traditional nanomaterials, peptide nanomaterials possess several advantages. They are biocompatible, easy to prepare and degrade, and resistant to high temperatures and chemical attacks. Therefore, they have become a hot research topic in electrochemical sensing and have been widely used in medical diagnosis [22], food safety monitoring [23], and environmental pollution detection [24,25]. Peptide-based sensors are usually responsible for supporting the self-assembled layer [26] and simulating specific target recognition elements [27]. They can be chemically modified by functional materials such as quantum dots [28,29], polymers [30], metal nanoparticles [31], or enzymes [32] to achieve the functionalization of peptides. Kyoung-Ik Min et al. modified tyrosine-rich peptide nanofibers with silver nanoparticles through biological reduction to form a universal nanoelectrode platform [33]. J. Castillo et al. immobilized phenylalanine dipeptide nanotubes modified with folic acid on graphene electrodes, enabling rapid label-free detection of various pathogens [34]. Meiling Lian et al. embedded the enzyme model horseradish peroxidase during the self-assembly process of Fmoc-FF and used it as a matrix for enzyme immobilization and cell adhesion, resulting in sensitive in situ monitoring of hydrogen peroxide released by HeLa cells [32]. The SAP-based biological interface is environmentally friendly and easy to prepare and can be integrated with various mediators, nanomaterials, and enzymes to monitor various cell biomolecules.

In this work, a cationic amphiphilic SAP Z23 was designed to disperse MWCNTs and construct composite electrodes for the sensitive detection of BPA. Z23 is rich in phenylalanine with a benzene ring structure and lysine with a positive charge. MWCNTs, functionalized with Z23 through simple electrostatic interaction and π-π stacking, could be well dispersed in water without chemical modifications on side walls. Furthermore, Z23 will form a hydrogel in phosphate-buffered saline (PBS), which enhances the adhesion of the modified MWCNTs. The preparation strategy for the Z23/MWCNTs nanocomposite and the modified electrode is straightforward. The electrostatic interaction between peptide Z23 and the negatively charged BPA integrated with the well-dispersed MWCNTs significantly improved the sensitivity and reduced overpotential in BPA detection compared with the bare GCE.

## 2. Materials and Methods

### 2.1. Material

Z23 (Ac-KFKFQFKFK-Am, where Ac means N-terminal acetylation and Am means C-terminal amidation; purity > 95%) was purchased from Shanghai Top-peptide Biotechnology Co. Ltd. MWCNTs (98%, external diameter 4–6 nm, length 10–20 μm) were purchased from Chengdu Organic Chemicals Co., Ltd., Chinese Academy of Sciences. BPA (99%) and ethanol (99.7%) were purchased from Shanghai Macklin Biochemical Co., Ltd. 10× PBS and PBS (pH 7.4) were purchased from Beijing Solarbio Science & Technology Co., Ltd.

### 2.2. Preparation of Z23/MWCNTs Nanocomposite

The Z23 stock solution was freshly prepared by dissolving the lyophilized Z23 powder in deionized water (18.2 MΩ cm) with vortex oscillation and ultrasonic treatment (Geneng G-030S ultrasonic cleaner, 180 W, 40 kHz, Shenzhen, China) until completely dissolved. MWCNTs were initially dispersed in deionized water with vortex oscillation and ultrasonic treatment, followed by the immediate addition of Z23 stock solution and deionized water. Then, the mixture was ultrasonicated for 15 min to disperse the Z23/MWCNTs. An aliquot of 10× PBS was injected into the dispersion to trigger the assembling of Z23 with another 2-min sonication, resulting in a 1× PBS ionic environment. The final concentrations of Z23 and MWCNTs were both 2 mg/mL. The mixture was incubated at room temperature for 1 h to ensure that the Z23 assembly was completed.

### 2.3. Preparation of the Modified Electrodes

First, the glassy carbon electrode (GCE, 3 mm diameter) was polished on a polishing cloth immersed with 0.05 μm alumina suspension and subsequently rinsed with deionized water, followed by ultrasonication in anhydrous ethanol and deionized water in sequence for 2–3 min and air-drying at room temperature. The treated electrode was placed in an electrolytic cell (10 mM potassium ferricyanide, 100 mM KCl). If the redox peak potential difference was about 59 mV in the cyclic voltammetry (CV) test, the surface of GCE was treated as clean, smooth, and neat. An aliquot (10 μL) of Z23/MWCNTs in PBS was placed on the surface of the pretreated GCE and dried at room temperature. The modified electrodes were preserved in PBS before testing. The modified electrode was labeled as Z23/MWCNTs/GCE.

### 2.4. Material Characterization

Diluted Z23 in PBS or dispersion of Z23/MWCNTs in water or PBS was placed on carbon film on the copper grid and stained with 1% uranium acetate. Images were collected by transmission electron microscopy (TEM) (Tecnai TF20, FEI, USA) at a working voltage of 200 kV. A rheometer (MCR302, Anton Paar, Austria) equipped with a 25 mm parallel plate system was used to measure the rheology of Z23 hydrogel (1 wt% in PBS) at 25 °C. Frequency scanning was performed at 0.1% strain in the linear viscoelastic region from 100 rad/s to 0.1 rad/s. An aliquot (10 μL) of Z23/MWCNTs in PBS was dropped onto a glassy carbon sheet and dried at room temperature. Scanning electron microscopy (SEM) (NanoSEM 450, NOVA, Israel) was used to characterize the morphology of the composite. For turbidity analysis, an aliquot of 10 μL 10× PBS was added to 90 μL of 10 mg/mL Z23 aqueous solution in a 96-well plate (Nunc, Denmark), and absorption kinetic was monitored at 313 nm with a plate reader (Infinite 200 Pro, Tecan, Switzerland) at room temperature. Meanwhile, 100 μL of 10 mg/mL Z23 aqueous solution and 100 μL of PBS were measured as controls. The circular dichroism (CD) spectrum was determined on a spectropolarimeter (J-720, Jasco, Japan) with a Starna quartz cuvette (1 mm path length) at room temperature.

### 2.5. Electrochemical Analysis

Electrochemical characterization of the composite electrode was conducted on an electrochemical workstation (CHI760E, Chenhua Instrument, China). The standard three-electrode system was applied with a modified GCE as the working electrode, a platinum wire as the counter electrode, and Ag/AgCl as the reference electrode. The electrochemical experiments were performed in PBS (pH 7.4) at room temperature. CV was used to explore the electrocatalytic performance of the sensor. Herein, the CV scanning potential of BPA electrocatalysis ranged from 0.1 V to 0.9 V, with a scanning rate of 0.1 V/s. The current-time (*i*-t) amperometric responses were collected at a constant working potential, with the successive injection of BPA solution into the electrolytic cell at equal time intervals. For BPA, an oxidation peak potential of 0.56 V was selected as the diffusion potential for the *i*-t test. The response time was calculated based on when the sensor reached 95% of its maximum current response value after adding the analyte. The linear response range was determined by linearly fitting the current value against the concentration after each addition of the analyte. The sensitivity $K/(\mu A\mu M^{-1}cm^{-2})$ was calculated based on the formula $k/R_{f}$, where *k* represents the fitted curve’s slope, and Rf represents the sensor’s effective surface area. The effective surface area was obtained following previous works [35]. The limit of detection (LOD) was estimated based on an S/N = 3.

## 3. Results and the Discussion

**Figure 1:**
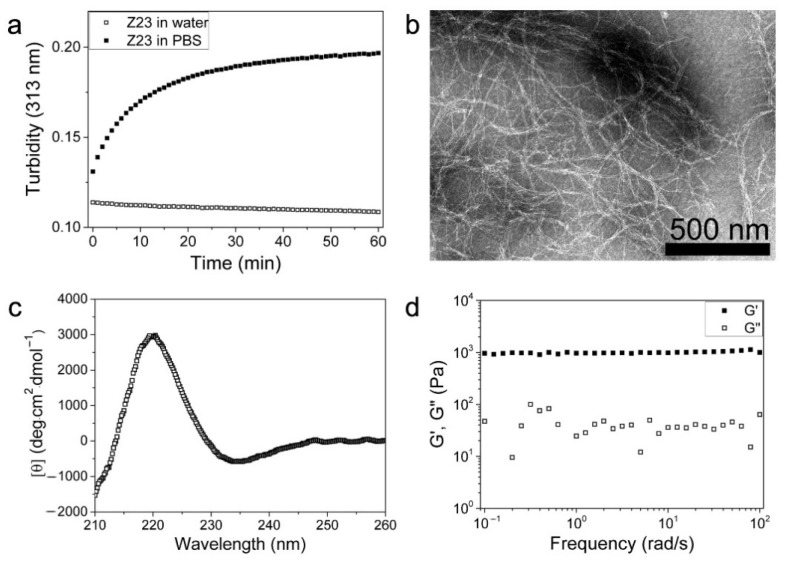
The self-assembly of Z23 in PBS. (**a**) Turbidity transformation of Z23 solution upon introducing PBS with that of Z23 in water as the control; (**b**) the TEM image of Z23 in PBS; (**c**) the CD spectrum of Z23 in PBS; (**d**) the frequency sweep of Z23 in PBS in the rheology measurement.

To disperse MWCNTs better, we designed the Z23 peptide with four phenylalanines to bind MWCNTs via a π-π interaction and four positively charged Lys for good solubility. Additionally, a polar uncharged Gln was included in the sequence to increase intermolecular hydrogen bonds without contributing to electrostatic repulsion or the hydrophobic interaction. The designed peptide was expected to bind MWCNTs and make the individual MWCNTs repel each other due to the strong positive charges, resulting in a good dispersion of MWCNTs in water. The self-assembly of Z23 was investigated first without the addition of MWCNTs. The turbidity of the Z23 solution increased over time and finally formed transparent hydrogels upon introducing PBS (Figure 1a). The salt-triggered self-assembling property was also observed in previously reported charged SAP systems [36,37]. Negative-stain TEM images showed that Z23 self-assembled in PBS to form networks of twisted and entangled nanofibers rich in β-sheet (Figure 1b,c), similar to other SAP nanofiber structures containing several aromatic residues [38,39]. The widths of the nanofibers were about 3 nm. The fact that the storage modulus (G′) was almost constant and considerably larger than the loss modulus (G″) in the swept frequency range indicated that Z23 could form hydrogels in PBS (Figure 1d).

**Figure 2:**
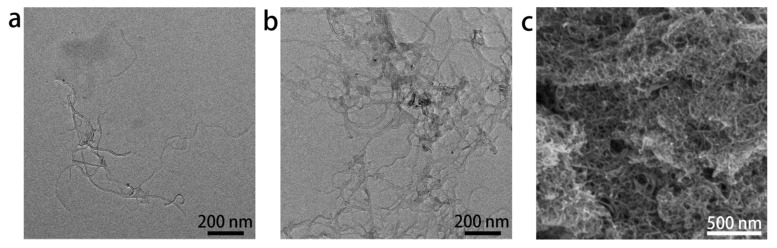
The microscopic morphology of the nanomaterials used to modify electrodes. TEM images of MWCNTs (**a**) and Z23/MWCNTs (**b**) in the suspension; (**c**) The SEM image of Z23/MWCNTs coated on a glassy carbon sheet.

In order to disperse MWCNTs with bath sonication, a certain amount of Z23 was applied. The obtained Z23/MWCNTs composite dispersion was visually inspected under intense light without apparent granular aggregates and could stand for at least two days without precipitation. When the mass ratio of Z23 to MWCNTs was 1:1, the MWCNTs could be dispersed at concentrations as high as 4 mg/mL. When the mass ratio was altered to 1:2, the highest concentration for the MWCNTs was 1.5 mg/mL. The dispersion effect was further examined using TEM, and most of the MWCNTs were observed as individual MWCNTs with diameters of 4–7 nm (Figure 2a), close to the information from the vendor (4–6 nm). By virtue of multiple phenyl groups and strong electrostatic repulsion, Z23 acted as a dispersant to prevent the agglomeration of MWCNTs. Upon introducing PBS into the dispersion, the unbonded peptide molecules assembled, and the Z23/MWCNTs composite formed networks (Figure 2b). The SEM image exhibited that the Z23/MWCNTs composite on a glassy carbon sheet had a three-dimensional (3D) porous structure (Figure 2c), which was favorable to diffusion and could provide plenty of adsorption sites for analytes. Taken together, the designed peptide Z23 could disperse MWCNTs and make composite gels with large specific surface areas simply and mildly.

**Figure 3:**
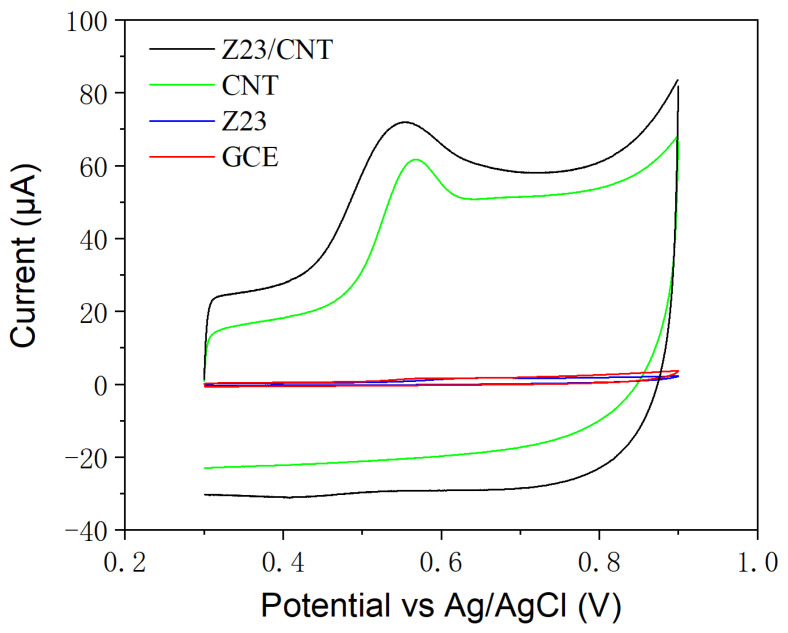
CVs of different modified electrodes in PBS buffer containing 15 μM BPA.

The as-prepared Z23/MWCNTs composite was applied to modify GCE for a highly sensitive electrochemical detection of BPA. Figure 3 shows the CV curves of different modified electrodes in PBS with 15 μM BPA. It may be seen that no obvious redox peaks are observed for the unmodified GCE, whereas the MWCNTs-modified GCE with a large surface area and good conductivity display an apparent oxidation peak as expected. Compared to MWCNTs/GCE, the oxidation overpotential of BPA on Z23/MWCNTs/GCE decreased slightly, and the peak current increased obviously. This excellent electrocatalytic performance might be attributed to three factors. First, MWCNTs have good electronic conductivity. Second, as an excellent gelator and dispersant for MWCNTs, Z23 effectively separated individual MWCNTs and supported MWCNTs in forming 3D networks, thereby increasing the effective surface area. Finally, the electrostatic attraction between protonated peptide Z23 and negatively charged BPA in a neutral environment resulted in the accumulation of BPA on the electrode surface. In addition, it is worth noting that GCE and CNT are conductors, while Z23 is not conductive and ionizes to form an electric double layer, which leads to a significant increase in capacitance after the composite modification of GCE.

**Figure 4:**
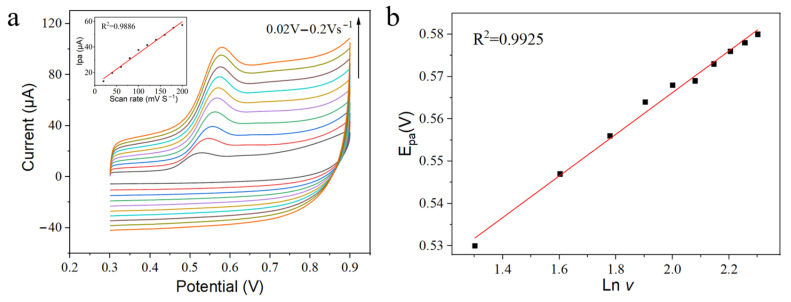
(**a**) CVs with different scan rates in the range of 0.02 to 0.2 Vs^−1^. (**b**) The fitting curve of the oxidation peak potential to BPA against the logarithm of the scan rate.

CV tests were conducted to investigate the electrochemical response of BPA on Z23/MWCNTs/GCE with varying scan rates. Figure 4a illustrates that the oxidation peak current (*I*_pa_) of BPA is directly proportional to the scan rate (*v*) between 0.02 Vs^−1^ and 0.2 Vs^−1^ (R^2^ = 0.9886), indicating that the oxidation of BPA on Z23/MWCNTs/GCE was a typical adsorption-controlled electrode process. In addition, the oxidation peak potential (*E_pa_*) shifts positively as the scan rate increases and exhibits a linear correlation with the logarithm of the scan rate (ln *v*) (Figure 4b). The Butler–Volmer equation $E_{pa}=E^{0}-\frac{RT}{\alpha nF}\ln\frac{RTk_{s}}{\alpha nF}+\frac{RT}{\alpha nF}\ln\;v$, obtained from Laville theory, could be applied to the adsorption-controlled and irreversible electrode process [33]. In this equation, $E^{0}/(V)$ represents half-peak potential; *R* is gas constant; *T* is absolute temperature; and *α* is the electron transfer coefficient, which usually takes an approximate value of 0.5. From the fitting slope of *E_pa_* to ln *v*, it could be inferred that the electron transfer number n of BPA electro-oxidation is 2, indicating a two-electron process [40].

**Figure 5:**
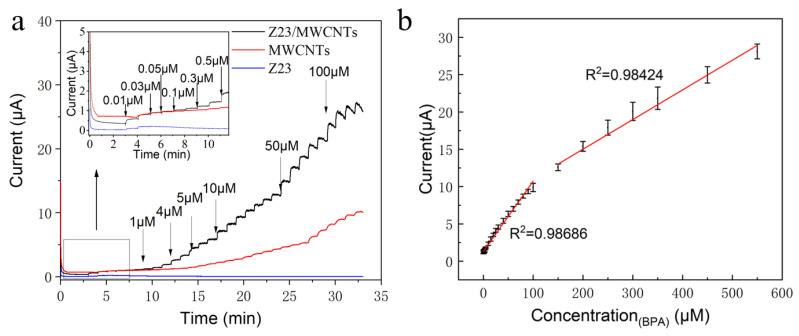
Amperometric responses (**a**) of different electrodes in PBS after successive additions of different concentrations of BPA at 0.56 V and the corresponding calibration curve (**b**) of the sensor constructed with Z23/MWCNTs.

The amperometric responses of the modified electrodes were investigated by successively adding BPA to PBS with magnetic stirring (Figure 5a). Z23-modified GCE had a negligible response due to its poor conductivity. However, the inclusion of Z23 in the composite significantly enhanced the electrocatalytic activity of the BPA oxidation compared with MWCNTs alone. The current response of Z23/MWCNTs/GCE was 3.24 times higher than that of MWCNTs/GCE. The enhanced BPA sensing performance of Z23/MWCNTs/GCE could be ascribed to its 3D network structure and numerous adsorption sites provided by Z23 on MWCNTs. The steady-state current of Z23/MWCNTs/GCE could be achieved within 2 s, resulting in rapid response to the variation of BPA concentration. Figure 5b showed the calibration curves of the response current to the BPA concentration, where piecewise linear regression was observed, in agreement with previous reports on BPA electrocatalysis. This phenomenon could be attributed to the effect of protonated Z23 peptide on the electrostatic adsorption of BPA in PBS. The adsorption effect was enhanced with increased BPA concentration, and the electroactive sites on the electrode surface might become saturated. The range covered by adsorbate material became uneven, hindering electron conduction, thus reducing the sensitivity and presenting two fitting linear ranges. The exact reason needs further study to reach a definite conclusion. Z23/MWCNTs/GCE exhibited a high sensitivity of 6.569 μAμM^−1^cm^−2^ in the low-concentration range between 0.01 μM and 100 μM with an R^2^ of 0.9929. The LOD was 1.28 nM (S/N = 3), lower than the previously reported value (Table 1). The enhanced performance of the Z23/MWCNTs/GCE sensor was attributed to the excellent electrocatalytic properties of carbon nanotubes and electrostatic adsorption of BPA by the four positively charged peptides Z23.

**Figure 6:**
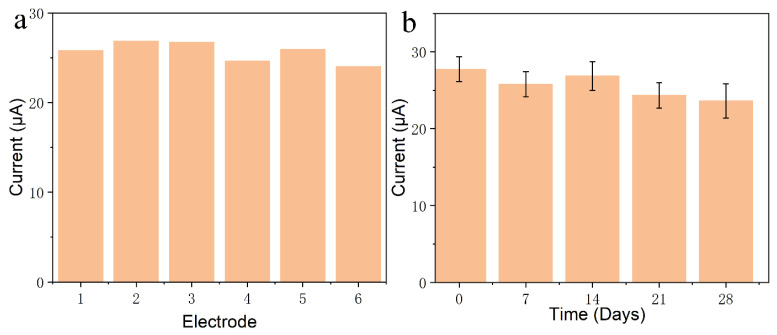
Stability testing. (**a**) Peak oxidation current of six sensors in PBS solution containing 15 μM BPA. (**b**) Changes in the peak oxidation current in a PBS solution containing 15 μM BPA for six sensors after 1–4 weeks in the refrigerator.

The reproducibility and long-term stability of the sensor were evaluated in order to examine the accuracy and practicability of this method. Figure 6a showed that six independent sensors were prepared by the same method, and the relative standard deviation (RSD) of current fluctuation obtained by measuring the peak current of 15 μM BPA was 5.17%, indicating excellent reproducibility and repeatability of the sensor. In addition, Figure 6b displays that the same batch of prepared electrodes was used to perform four measurements at one-week intervals. When the electrodes were not in use, they were immersed in PBS and placed at 4 °C. The response current remained at 82.65% of the initial value, indicating that the modified electrode had good stability. Accordingly, Z23/MWCNTs/GCE could be used for trace detection of BPA in practical applications.

## 4. Conclusions

In this work, we designed the positively charged peptide Z23 to disperse MWCNTs, and the resulting composites were applied to modify GCE to construct an electrochemical sensor for BPA detection. The composite material had a three-dimensional network structure, which could increase the electroactive area, accelerate electron transfer between electrode interfaces, and reduce overpotential compared with the bare GCE. The Z23/MWCNTs/GCE sensor has the advantages of high sensitivity, wide linear range, low detection limit, long-term stability, and environmental friendliness, and is easy to prepare. Therefore, it has a potential application value in food safety and environmental monitoring.

## Figures and Tables

**Table 1:** Performance comparison of BPA detection with other sensors.

Modified Electrode	Detection Method	Linearity Range (μM)	Limit of Detection (nM)	Response Time (s)	References
MWCNTs/CuFe_2_O_4_/GCE	DPV	0.01–120	3.2	—	[41]
MWCNTs-PEI/GCE	DPV	0.01–50	3.3	—	[42]
CMK-3/nano-CILPE	LSV	0.2–150	50	—	[43]
MWCNTs-COOH/GCE	LSV	0.001–10	5	6	[44]
MWCNTs-OH/GCE	FIA	0.032–0.76	2.57	<10	[45]
Tyr-DAPPT-rGO/GCE	*i*-t	0.001–38	0.35	<10	[46]
CB/f-MWCNTs/GCE	*i*-t	0.1–130	80	—	[47]
MWCNT/AuNP/GCE	DPV	0.01–0.7	4	<10	[48]
Z23/MWCNTs/GCE	*i*-t	0.01–100	1.28	2	This work

PEI polyethyleneimine; CMK-3 ordered mesoporous carbon; CILPE carbon ion liquid paste electrode; AuNPs gold nanoparticles; -COOH carboxylation; -OH hydroxylation; Tyr tyrosinase; DAPPT 1,3-bis(4-amino-1-pyridine) propane tetrafluoroborate ionic liquid; rGO reduced graphene oxide; FIA flow injection amperometric method; LSV linear sweep voltammetry.

## Data Availability

Data are contained within the article.
